# Health-Related Physical Fitness of Romanian University Students: The European Fitness Badge Approach

**DOI:** 10.3390/healthcare13161966

**Published:** 2025-08-11

**Authors:** Vlad Adrian Geantă, Viorel Petru Ardelean, Corina Dulceanu, Claudiu Bulzan, Patricia Roxana Forț, Borko Katanic, Karuppasamy Govindasamy, Francisco Campos, Ricardo Gomes, Vasile Emil Ursu, Ovidiu Gheorghe Șerban

**Affiliations:** 1 Faculty of Physical Education and Sport, Aurel Vlaicu University of Arad, 310330 Arad, Romania; corina.dulceanu@yahoo.com (C.D.); claudiubulzan@yahoo.com (C.B.); pantarpatriciar@yahoo.com (P.R.F.); ovi13r@yahoo.com (O.G.Ș.); 2 Montenegrin Sports Academy, 81000 Podgorica, Montenegro; 3 Department of Sports Recreation and Wellness, Symbiosis International (Deemed University), Hyderabad Campus, Modallaguda, Nandigama, Rangareddy Dist., Hyderabad 509217, Telangana, India; gowthamadnivog@gmail.com; 4 Coimbra Education School, Polytechnic University of Coimbra, Rua da Misericórdia, Lagar dos Cortiços, S. Martinho do Bispo, 3045-093 Coimbra, Portugal; francicampos@esec.pt (F.C.); rimgomes@esec.pt (R.G.); 5 SPRINT-Sport Physical Activity and Health Research & Innovation Center, Polytechnic University of Coimbra, Rua da Misericórdia, Lagar dos Cortiços, S. Martinho do Bispo, 3045-093 Coimbra, Portugal; 6 Department of Physical Education and Sport, Faculty of Law and Social Sciences, University “1 Decembrie 1918” of Alba Iulia, 510009 Alba Iulia, Romania; vasile.ursu@uab.ro

**Keywords:** European Fitness Badge, physical fitness, body composition, university students, health

## Abstract

**Background/Objectives:** Physical fitness is a key determinant of health in university students, a population at risk of sedentary behaviors and lifestyle-related health issues. The European Fitness Badge (EFB) provides a comprehensive assessment of fitness parameters and individualized feedback. This study aimed to evaluate the physical fitness of Romanian university students using the EFB to examine the effects of activity profile, sex, age, and academic major and to associate physical fitness with body composition indices, including Body Mass Index (BMI) and A Body Shape Index (ABSI). **Methods:** A cross-sectional study was conducted with 180 university students (43.33% male; age 18–53 years). Participants were categorized into two fitness profiles: Basic (TP1) and Advanced (TP2). Physical fitness was assessed through standardized EFB protocols measuring endurance, strength, flexibility, coordination, and overall fitness. Independent *t*-tests, one- and two-way ANOVA, and Pearson correlations were used to analyze differences and associations. **Results:** TP2 participants scored significantly higher in endurance, strength, coordination, and overall fitness, Males outperformed females in strength and coordination. Strength decreased with age, particularly in the oldest group. The sports students demonstrated superior fitness compared to peers in other majors. Two-way ANOVA revealed significant interactions between profile and sex for strength and overall fitness. BMI and ABSI were negatively correlated with physical fitness, in strength, coordination, and overall fitness. **Conclusions:** The EFB proved effective in differentiating physical fitness profiles. Demographic factors and body composition influenced fitness outcomes, underscoring the need for targeted, profile-based physical activity strategies specifically adapted to university curricula and extracurricular sport programs. However, the findings should be interpreted in light of the study’s geographic limitation, as the sample included only Romanian university students.

## 1. Introduction

Physical fitness is a key determinant of overall health and well-being, encompassing multiple components [1,2,3]. It is widely recognized as a key predictor of obesity, with some investigations suggesting that fitness level could be more important than body weight in determining overall health and well-being [4,5,6].

University students constitute a key demographic for investigating physical fitness, as the transition to independent living often triggers substantial lifestyle changes [7]. This phase is frequently associated with increased sedentary behavior, reduced physical activity, and poor dietary habits, all negatively impacting on health and fitness levels [8,9,10], highlighting the need for structured interventions [11].

Furthermore, variations in fitness levels based on sex, age, and field of studies (academic major) have been observed, suggesting that demographic factors play an important role in shaping students’ physical profile [2,12]. Recent research shows that, compared to their male counterparts, female students, despite meeting step count recommendations, have fewer time spent in moderate-to-vigorous physical activity [13,14,15].

The Eurobarometer on sports and physical activity [16] points out that 62% of the Romanian population do not engage in any form of exercise or sports activity, despite numerous governmental programs and strategies aimed at promoting mass sports.

Recent studies show that sociodemographic factors such as age, income, and education level continue to shape the physical activity patterns of Romanian adults, with post-pandemic periods showing a particularly pronounced decrease in activity levels, further exacerbating the gap in sports participation [17]. Moreover, some research highlights that health choices and attitudes towards physical activity in Romania, especially among children and young people, are shaped by the quality of health education and the environment in which they grow up, with limited access to either school-based or community-based structured health-promoting activities [18].

High levels of physical activity and exercise are positively correlated with lower BMI and overall physical health [19,20], although with some disparities across different demographic groups, suggesting that policy efforts may not be equally effective for all populations [21]. Beyond BMI, physical activity also contributes significantly to cardiovascular health and psychological well-being [22,23]. Sedentary behavior, however, remains a persistent risk factor across populations [24].

Studies suggest that structured exercise programs tailored to demographic characteristics can significantly improve physical fitness levels in university populations [25,26]. A systematic review examining physical activity and fitness among university students highlights that, while overall activity levels may be satisfactory, they vary due to cultural and educational system differences, further emphasizing the need for targeted interventions [2].

To evaluate various components of physical fitness, multiple standardized assessment tools have been developed. Among these, the European Fitness Badge (EFB) is a widely recognized tool designed to assess endurance, strength, flexibility, coordination, and overall fitness, while providing personalized feedback [27,28,29]. Developed as part of a European Erasmus+ Sport “Cooperation Partnership” project, the EFB incorporates a series of structured tests aimed at delivering a multidimensional evaluation of physical fitness. Unlike other test batteries, the EFB assesses and categorizes the individuals based on their fitness level, offering targeted recommendations for improvement. A key innovation of the EFB is its ability to generate real-time, individualized feedback delivered via email, enhancing user engagement and supporting sustained physical activity behaviors. Its structured approach makes it a valuable tool for identifying areas that require intervention and promoting active lifestyles among university students. Recent studies highlighted the effectiveness of the EFB in assessing Health-Enhancing Physical Activity (HEPA) across populations. For instance, research by Klemm et al. demonstrated the EFB’s utility as a diagnostic tool within HEPA framework, emphasizing its role in promoting active lifestyles among university students [29].

Beyond the EFB, several other standardized fitness assessment batteries exist to measure physical fitness components. Among the most widely used are: FitnessGram, designed primarily for youth populations in the United States [30,31]; EuroFit, widely implemented across Europe [32,33,34]; and Alpha Fit, focused on health-related fitness in children and youth [35]. Additionally, sport-specific protocols have been developed to assess physical fitness in athletic populations. For example, Sgrò et al. [36] investigated the impact of sport practice on health-related fitness components (endurance, strength) in youth volleyball and water polo athletes.

Research focusing on the EFB and its applicability in university settings is scarce. The EFB not only provides personalized feedback but also serves as a valuable instrument to identify areas requiring intervention, thereby promoting an active lifestyle among university students. Recent research demonstrated the applicability of the EFB in several adult populations across Europe. The study of Klemm et al. [28] examined data from 6019 adults. Despite its growing use, few studies have specifically focused on university populations, and even fewer have explored country-specific applications such as Romania. This study aims to bridge that gap by applying the EFB within a Romanian university context.

This lack of data is particularly relevant given the ongoing discussion about potential disparities in physical fitness profiles between young adults in Eastern and Western Europe, influenced by socioeconomic, cultural, and educational differences. Several studies suggest that physical activity engagement, health literacy, and access to structured fitness programs are generally lower in Eastern Europe, which may negatively affect fitness outcomes in youth and young adults [17,28]. In addition, a low level of physical fitness in university students has been associated with an increased risk of long-term health conditions, including metabolic syndrome, cardiovascular disease, and premature mortality [4,24,37]. Therefore, applying the EFB in the Romanian university context is not only novel but also essential to better understand region-specific health risks and to inform targeted strategies for physical activity promotion in this population.

Evidence suggests that physical fitness is influenced by several demographic factors, including gender, age, and field of academic study. Male students tend to show higher levels of cardiorespiratory endurance and muscular strength, while female students may perform better in flexibility tests [37]. Age-related differences also emerge within university populations, particularly in relation to lifestyle patterns and engagement in regular physical activity [38]. Additionally, students enrolled in sports science or physical education programs typically exhibit higher fitness levels than their peers in non-sport-related departments, likely due to curriculum demands and personal interest in physical activity [39]. These factors should be considered when interpreting fitness assessment data in university settings.

By addressing these gaps, the present study aims to examine how university students’ physical fitness assessed through the EFB varies according to test profile (TP1—Basic; TP2—Advanced), sex, age, and academic major. In addition, it explores whether body composition indices, such BMI and ABSI, are associated with fitness performance.

Based on existing evidence, we expect that students assigned to TP2 will exhibit significantly higher scores across fitness characteristics (endurance, strength, coordination, flexibility) compared to those in TP1. We also anticipate that male students will perform better than females in strength and coordination; that strength levels will decline with age; and that students enrolled in sports-related majors will demonstrate superior physical fitness. Furthermore, we hypothesize that both BMI and ABSI will be negatively correlated with performance in physical fitness components, particularly strength and overall fitness.

Rather than validating the EFB protocol itself, this study applies it in a Romanian academic context, with the aim of providing practical, context-sensitive insights into the physical fitness characteristics of university students. We consider that these findings may support the development of tailored, profile-based strategies to enhance physical activity and health promotion in higher education environments.

## 2. Materials and Methods

### 2.1. Participants

This study employed a cross-sectional observational design, conducted among Romanian university students, to assess physical fitness using the EFB. The sample was selected using a convenience sampling strategy, based on voluntary participation in scheduled fitness assessments conducted at the university sports facilities.

A total of 180 students (78 males, 43.33%; 102 females, 56.66%) aged between 18 and 53 years (M ± SD = 23.53 ± 7.38) participated in the study. Age distribution was as follows: 18–25 years (n = 141; 78.33%), 26–35 years (n = 24; 13.33%), 36–45 years (n = 11; 6.11%), and over 45 years (n = 4; 2.22%). It is important to note that the age distribution was skewed toward younger participants, which may limit the generalizability of age-related findings due to low representation in older age groups.

Participants were enrolled in diverse academic programs: Sports (n = 35; 19.4%), Social Work (n = 36; 20.0%), Psychology (n = 16; 8.9%), Engineering (n = 33; 18.3%), Computer Science (n = 26; 14.4%), Accounting (n = 18; 10.0%), and Pedagogy (n = 16; 8.9%). Although not equal across disciplines, the distribution allowed comparative analysis between sports-related and non-sports academic majors.

Based on the EFB criteria, students were classified into two fitness profiles: Test Profile 1 (TP1—Basic), corresponding to lower physical activity levels, and Test Profile 2 (TP2—Advanced), representing more active individuals. The TP1 group included 93 students (51.7%; 19 males and 74 females), while the TP2 group comprised 87 students (48.3%; 59 males and 28 females).

Initially, a total of 252 university students were considered for inclusion in the study. Of these, 28 students (11.1%) were excluded due to medical contraindications identified through the Physical Activity Readiness Questionnaire (PAR-Q) [40]. An additional 44 students (17.5%) either declined participation, withdrew consent, or failed to attend testing sessions, resulting in a final participation rate of 71.4%. The final sample thus comprised 180 students who met all inclusion criteria and completed the testing protocol.

**Figure 1:**
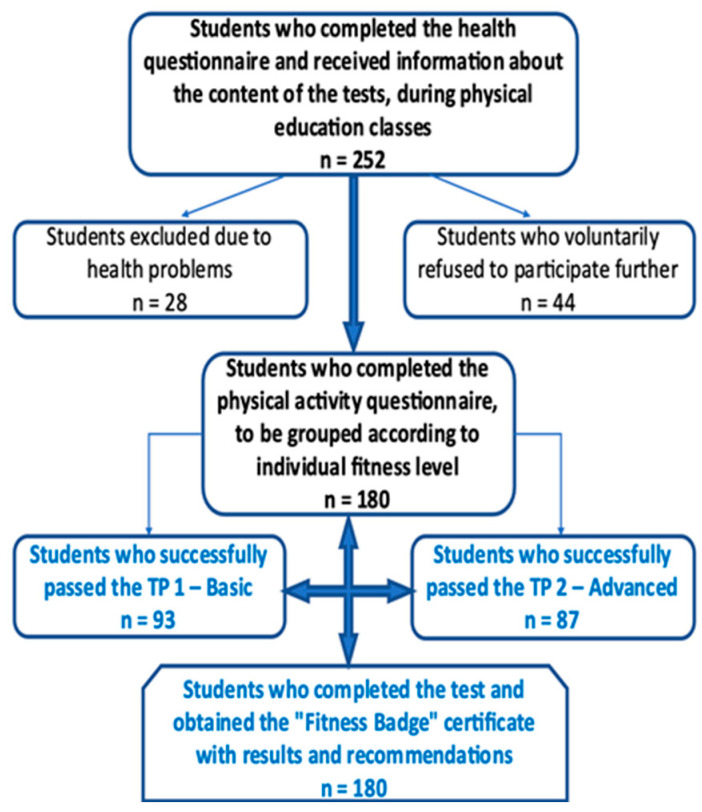
Flowchart of participant inclusion and categorization based on health screening (PAR-Q), voluntary consent, and EFB profile classification.

An a priori sample size calculation was performed using G*Power 3.1, based on a two-tailed *t*-test with α = 0.05, power = 0.80, and an expected medium-to-large effect size (Cohen’s d = 0.61–0.65). The analysis indicated a minimum sample of 128 participants. To allow for subgroup analysis and potential dropout, a total of 180 students were included. Participant eligibility and group classification were determined based on the EFB assessment system, as illustrated in Figure 1.

All participants provided written informed consent prior to their inclusion in the study, in accordance with the principles outlined in the Declaration of Helsinki. The study protocol was reviewed and approved by the Ethics Committee of the Faculty of Physical Education and Sport, Aurel Vlaicu University of Arad (Protocol No. 135a/3 February 2021).

### 2.2. Measurements and Tests

Prior to physical fitness assessment, all participants completed the Physical Activity Readiness Questionnaire (PAR-Q) [40], a validated screening tool consisting of seven items aimed at identifying potential health risks associated with physical activity. If the students answer yes to one question or more, they are excluded due to health problems.

After this, they answered the Activity Questionnaire, available on the EFB [41], which classified and allocated the university students to TP1 or TP2 profiles. This classification considered both the PAR-Q results and participants’ self-reported exercise habits [41], as outlined in the EFB framework for physical activity readiness and test suitability.

However, it is important to note that the final profile allocation was based primarily on self-reported activity level and not confirmed by physical test performance. Consequently, a number of participants (n = 20 in TP1 and n = 21 in TP2) did not reach the minimum score threshold for their assigned test profile during fitness assessment, revealing a discrepancy between perceived and actual fitness levels.

The EFB is a standardized fitness assessment protocol developed according to HEPA principles, specifically designed for adults aged 18 to 65 years. It provides a multidimensional evaluation of physical fitness, including endurance, strength, coordination, and flexibility, alongside anthropometric measurements such as weight, height, and waist circumference, allowing the calculation of BMI and A Body Shape Index (ABSI), a composite index to better evaluate obesity-related health risks [42,43,44].

Endurance was assessed using the Danish step test; muscular strength was evaluated through push-ups, plank, and jump-and-reach tests; coordination with flamingo balance and backward walking tests; and flexibility using the sit-and-reach test [41].

The performance score was based on established normative data from previous research, ensuring that evaluation thresholds were appropriately age-adjusted [41].

In TP1, participants under the age of 40 were required to achieve a minimum of 11 points to meet the basic fitness standard, with age-related reductions applied for older participants.

For TP2, scores were classified in two levels, for scores equal to or greater than 11 points (Approved) and for scores of 15 points or higher (Advanced). The maximum score achievable was 20, consistent with reference values employed in international fitness assessment studies (Table 1) [41].

### 2.3. Procedures

Testing took place during scheduled Physical Education classes in the university’s sports facilities, under the supervision of certified researchers. In addition, certain testing sessions were conducted under the direct supervision of certified instructors, in collaboration with the original developers of the European Fitness Badge (EFB), ensuring fidelity to the standardized protocol.

After completing the PAR-Q and Activity Questionnaire, participants were grouped into TP1 or TP2. The testing procedures followed the standardized EFB protocol. Before starting, participants met the researchers and were guided on how to perform each test safely and accurately. All physical assessments and anthropometric measurements were completed in a single session. Short rest intervals were incorporated between tests to minimize fatigue effects and maintain the quality of performance. Testing was conducted indoors, in a well-ventilated, temperature-controlled sports hall under consistent and standardized conditions. Environmental or weather-related factors did not influence any of the testing sessions.

Although the EFB protocol allows flexibility in sequencing, the tests were generally administered in an alternating order of intensity (e.g., endurance, coordination, strength, flexibility) to prevent cumulative fatigue. The step test, used to assess endurance, was limited to a maximum of 2 min and 40 s in TP1 and 6 min (or a 2 km walk) in TP2. All performance scores were calibrated based on age and sex, in accordance with the normative scoring tables provided in the official EFB Instructor’s Handbook.

Upon completion, participants received an official “Fitness Badge” certificate, including their profile, test results, and personalized recommendations.

### 2.4. Statistical Analysis

Descriptive statistics, with measures of central tendency (M ± SD), were calculated to summarize participant’s physical fitness performance. For the inferential analysis, after checking the normality assumption [44], an independent sample *t*-test was employed to compare physical fitness between profile and sex subgroups. The one-way ANOVA, after checking normality and homogeneity assumptions [45], was used to assess differences across age groups and academic majors. Where appropriate, post hoc comparisons were conducted using the Bonferroni correction [45]. Two-way ANOVA was applied to examine interaction effects between sociodemographic characteristics (profile, sex, age group, and academic major) and physical fitness parameters.

For the *t*-test, Effect Size (ES) calculations were performed through Cohen’s *d* reference values, according to O’Donoghue [46]: small (*d* < 0.20); moderate (0.20 ≤ *d* < 0.80); large (*d* ≥ 0.80). For the one-way ANOVA, ES was calculated according to the eta squared (*η*^2^) reference values [47]: small (*η*^2^ ≤ 0.05); medium (0.05 < *η*^2^ ≤ 0.25); high (0.25 < *η*^2^ ≤ 0.50); very high (*η*^2^ > 0.50).

Pearson correlation was conducted to explore statistical associations between BMI and ABSI with physical fitness parameters. To assess the magnitude and direction of the associations, the correlation (*ρ*) was performed following the validation of the normality assumption [48]. The normality of each univariate variable was evaluated using the Kolmogorov–Smirnov test. When normality was not confirmed we relied on the Central Limit Theorem (CLT), given the large sample size (n = 180) justifying the use of Pearson correlation. This is a widely accepted approach in applied health sciences when sample sizes are sufficiently large [44]. Intensity of the associations between the variables was classified according to Hopkins’ scale [49]: very weak (*ρ* < 0.1); weak (0.1 < *ρ* ≤ 0.3); moderate (0.3 < *ρ* ≤ 0.5); strong (0.5 < *ρ* ≤ 0.7); very strong (0.7 < *ρ* ≤ 0.9); almost perfect (*ρ* > 0.9); perfect (*ρ* = 1.0).

The inferential statistics analyses were conducted using IBM SPSS version 28.0 (IBM Corp., Armonk, NY, USA), for a significant level of 5% (*p* < 0.05) for all tests.

## 3. Results

TP1 presents a score of 2.80 ± 0.42 in endurance, 7.87 ± 0.99 in strength, 2.64 ± 0.60 in flexibility, 5.15 ± 0.55 in coordination, and 10.86 ± 0.88 in overall fitness (Table 2). The TP2 presents higher values: 3.00 ± 0.01 in endurance, 9.00 ± 0.01 in strength, 2.72 ± 0.56 in flexibility, 5.65 ± 0.72 in coordination, and 12.66 ± 3.67 in overall fitness. Since a higher score reflects a higher level of physical fitness capacity, participants with the Advanced profile consistently present better results. Nevertheless, in both groups, a small number of participants (TP1: n = 20; TP2: n = 21) failed to meet the profile-specific fitness performance thresholds, despite being categorized according to their self-reported activity level. These inconsistencies are discussed further in the Discussion section. In total, for characterization of the physical fitness of the university students, the score of endurance is 2.90 ± 0.31, 8.41 ± 0.90 in strength, 2.68 ± 0.58 in flexibility, 5.39 ± 0.68 in coordination, and 11.73 ± 2.77 in overall fitness.

Comparing the university student profiles (TP1 vs. TP2), significant differences were found for endurance (*t* = −4.260; *p* = 0.001; *d* = −0.635; moderate ES), strength (*t* = −10.610; *p* = 0.001; *d* = −1.584; large ES), coordination (*t* = −5.263; *p* = 0.001; *d* = −0.785; moderate ES), and overall fitness (*t* = −4.588; *p* = 0.001; *d* = −0.684; moderate ES). These differences are not only statistically significant but also practically meaningful, as shown by the large effect size for strength (*d* = −1.584) and moderate effect sizes for other components. This supports the relevance of the EFB as a functional diagnostic tool capable of capturing real, actionable differences in physical capacity among students.

Significant differences were observed between male and female participants for strength (*t* = 6.754; *p* = 0.001; *d* = 1.016; large ES) and coordination (*t* = 3.909; *p* = 0.001; *d* = 0.588; moderate ES), with males obtaining higher scores in both parameters (Table 3). Age group differences occurred only for strength (*F* = 3.260; *p* = 0.023; *η*^2^ = 0.058; medium ES). However, Bonferroni post hoc comparisons did not reveal statistically significant differences between specific age groups. Given the conservative nature of the Bonferroni test and the small sample size in some age categories, particularly in older groups, these differences may have lacked sufficient statistical power to reach significance. Significant differences were also found based on students’ academic major for endurance (*F* = 2.724; *p* = 0.015; *η*^2^ = 0.003; small ES), strength (*F* = 7.640; *p* = 0.001; *η*^2^ = 0.089; medium ES), coordination (*F* = 2.702; *p* = 0.016; *η*^2^ = 0.003; small ES), and overall fitness (*F* = 2.335; *p* = 0.034; *η*^2^ = 0.001; small ES). To clarify these differences, the results of the post hoc Bonferroni tests are reported below. Students from the Sports major had significantly higher scores in strength compared to those from Social Care (*p* = 0.001), Psychology (*p* = 0.005), Accounting (*p* = 0.013), and Pedagogy (*p* = 0.001). In terms of coordination, Sports students also outperformed those from Social Care (*p* = 0.049) and Pedagogy (*p* = 0.032). No statistically significant differences were found between academic majors for endurance, flexibility, or overall fitness after correction (*p* > 0.05 for all pairwise comparisons).

To analyze and understand the combined effects of students’ characteristics (TP, sex, age, and academic major) with their physical fitness parameters, a two-way ANOVA was applied (Table 4). The results showed a significant interaction for strength (*F* = 8.575; *p* = 0.004) and overall fitness (*F* = 4.174; *p* = 0.043), indicating that both TP and sex significantly and positively influence these physical fitness parameters. Further analysis revealed that the interaction between TP and sex had a moderate effect size for both strength (*η*^2^ = 0.066) and overall fitness (*η*^2^ = 0.056), suggesting that the combination of these factors explained a meaningful proportion of variance in performance. Specifically, male students with a test profile achieved the highest scores, while female students without a test profile scored the lowest in both strength and overall fitness.

Lastly, Pearson correlation coefficients (Table 5) reveal a significant negative correlation between BMI and strength (*p* = −0.214; *p* = 0.004; weak intensity), coordination (*ρ* = −0.277; *p* = 0.001; weak intensity), and overall fitness (*p* = −0.175; *p* = 0.019; weak intensity) and between ABSI and overall fitness (*p* = −0.264; *p* = 0.001; weak intensity). These results suggest that higher BMI and ABSI are associated with lower levels of overall fitness and of strength and coordination in the case of BMI. In contrast, Test Profile (TP) showed strong positive associations with strength (*p* = 0.623; *p* < 0.001; strong intensity) and moderate associations with endurance (*p* = 0.304; *p* < 0.001; moderate effects), coordination (*p* = 0.367; *p* < 0.001; moderate effect), and overall fitness (*p* = 0.325; *p* < 0.001; moderate effect). These findings highlight the relevance of structured training or performance testing in predicting better physical fitness levels. Sex was significantly correlated with strength (*p* = −0.452; *p* < 0.001; moderate-to-strong intensity) and coordination (*p* = −0.281; *p* = 0.001; weak intensity), suggesting that male participants tended to perform better in these domains. No significant associations were observed between sex and other components. Age was weakly but significantly negatively correlated with strength (*p* = −0.207; *p* = 0.005; weak effect) and positively with overall fitness (*p* = 0.160; *p* = 0.032; weak effect), suggesting a minor influence of age on performance. Academic major showed weak negative associations with strength (*p* = −0.213; *p* = 0.004; weak effect) and overall fitness (*p* = −0.149; *p* = 0.046; weak effect), possibly reflecting differences in physical training exposure across fields of study. No significant correlation was observed between BMI and flexibility or endurance nor between ABSI and any individual fitness components such as endurance, strength, coordination, or flexibility (all *p* > 0.05), indicating negligible or very weak associations.

## 4. Discussion

This study revealed several significant findings regarding the relationships between profile, sex, age, and academic major in university students in relation to physical fitness levels. Differences were observed between participants with different fitness levels, with those having an Advanced profile (TP2) outperforming those with a Basic (TP1) one in endurance, strength, coordination, and overall fitness. Male participants demonstrated significantly higher levels of strength and coordination compared to females. Age was also a significant variable, with strength decreasing as age increased, with significant differences found between the youngest (19–25 years) and oldest (over 45 years) age groups. Academic major also influenced physical fitness, with sports students performing better than peers from non-sports majors. The results also showed that both TP and sex significantly influence the strength and overall fitness of university students.

The present research further showed that BMI was negatively correlated with strength, coordination, and overall fitness and ABSI with overall fitness. This distinction is particularly important because ABSI incorporates waist circumference relative to height and weight, thus providing a more sensitive measure of visceral adiposity compared to BMI, which only considers weight and height. Visceral fat is known to have a stronger negative impact on metabolic health and physical fitness outcomes than general body mass alone [50]. Therefore, the significant correlation between ABSI and overall fitness emphasizes the clinical relevance of assessing body composition beyond BMI, especially in young adult populations [51]. Recent evidence also suggests a complex, sometimes paradoxical, relationship between ABSI and cardiometabolic health, warranting further investigation in diverse cohorts [52]. It is important to interpret these results in the context of Romanian-specific cultural and educational factors. While Romania has implemented various national strategies to promote physical activity, data from the Eurobarometer suggest that 62% of Romanians still do not engage in any form of physical exercise [16]. Moreover, limited access to structured fitness programs in non-sport academic areas, combined with low-quality health education in schools and universities [17,18], may contribute to the reduced fitness levels observed in non-sports majors. Therefore, sociocultural and systemic educational factors should be acknowledged as key contextual variables influencing the findings.

Overall, our findings are in line with those of Klemm et al. [27], who found that physical fitness in students is significantly impacted by age, sex, body composition, and level of engagement in physical activity. It should be noted that male participants demonstrated higher levels in fitness parameters compared to females, aligned with findings from earlier studies [27,53,54].

These results may be partly explained by sedentary behaviors of university students, who often spend extended periods attending classes and studying. This is supported by a review study [55] that identified a high prevalence of sedentary behavior among university students across diverse contexts. The increasing sedentary lifestyle among young people poses numerous health risk [56]. Also, the COVID-19 pandemic has further influenced physical activity, introduced restrictions [57,58,59,60], but also led to increased participation in outdoor activities like hiking, cycling, and walking [61,62].

Male students outperformed females in strength and coordination, while Advanced profile individuals showed superior endurance, strength, coordination, and overall fitness scores. These results align with general trend in fitness, where men prioritize muscle mass development while women focus on mobility and endurance-based activities. These gender differences in coordination may be partially explained by biomechanical factors, such as a wider pelvis and increased Q-angle in females, which can influence movement control and stability [63]. Additionally, sociocultural aspects, including less exposure to structured physical training or sports requiring complex coordination, may also play a role in shaping motor performance patterns in women.

To better understand the impact of these findings beyond statistical significance, it is important to consider their practical relevance. The effect sizes reported, particularly the large effect for strength and moderate ones for coordination and overall fitness, further confirm that these are meaningful differences with implications for health promotion and individualized training. The EFB protocol highlights these distinctions and helps tailor exercise recommendation accordingly. Differences in balance and coordination were also notable. Given the nature of the EFB protocol, which emphasizes lower-body engagement, male participants who reported engaging in recreational sports such as soccer during their leisure time demonstrated slight advantages in these areas. Furthermore, the test effectively distinguished between individuals engaging in regular, intensive training and those leading sedentary lifestyle, confirming its validity as a diagnostic tool.

These findings suggest that the EFB questionnaire-based classification may overestimate actual fitness levels in some individuals. The discrepancy between subjective and objective measures was especially evident among sports majors, pointing to the need for more accurate self-assessment and structured evaluation tools. Meanwhile, students from more sedentary fields such as psychology or social work showed lower performance, emphasizing the value of tailored physical activity interventions for non-sport academic disciplines.

Regarding the effect of age on strength, significant differences were observed, with younger participants outperforming older ones. These differences are likely linked to behavioral and lifestyle changes rather than physiological aging. Even with a young adult population, such as university students, lifestyle patterns tend to shift as academic years progress. Kljajević et al. [64] found that physically active students demonstrate significantly better physical fitness, particularly in strength and endurance, than sedentary peers. Increased academic workload, reduced participation in structured physical activity, and more sedentary behavior over time may contribute to reduced muscular performance even in early adulthood. These behavioral changes may partially explain the observed differences in strength, despite the relatively narrow age range our sample. However, the small number of participants in the oldest subgroup (n < 5) limits the generalizability of this finding and reduces the statistical power for between-group comparisons. Future research with a more balanced age distribution and objective measures of physical activity is recommended.

An unexpected finding was the absence of significant flexibility differences between age or sex groups. This contrasts with previous research, such as that of Li et al. [65], who found higher flexibility levels among female university students. A possible explanation may lie in the limited sensitivity of the sit and reach test, which primarily assesses hamstring and lower-back flexibility. In addition, the narrow age range and low variability in flexibility scores may have reduced the power to detect group differences. The results highlight the need for more sensitive and comprehensive flexibility assessments in similar populations.

The results of this research highlight the importance of promoting physical activity among students to improve their physical fitness [66,67,68,69]. This should serve as the first step in emphasizing the significance of physical fitness for overall health, with a call for collective involvement in taking further necessary steps toward this goal. Practical implications suggest that these findings could be useful in developing strategies to improve the physical fitness among teenagers. Overall, they emphasize the necessity of structured fitness programs and ongoing assessments tools to promote health and well-being. The EFB test provides an effective method for evaluating and improving physical fitness among university students and broader populations.

### 4.1. Limitations

This study was limited to university students, restricting the generalizability of the findings to the broader population. As a cross-sectional investigation, it captures a single point in time and therefore precludes causal inferences or the assessment of temporal trends. Selection bias may be present, as university students tend to be more physically active than the general adult population [3,69,70,71]. Additionally, components of the EFB assessment rely on self-reported data, which may introduce inaccuracies due to under- or overreporting of physical activity. This may include recall bias or social desirability effects, particularly in the Activity Questionnaire.

The cultural and academic environment of the study sample may also influence fitness behaviors, potentially limiting the applicability of findings to other educational or regional contexts [69]. For example, norms and expectations related to physical activity may differ across countries, potentially introducing cultural bias when generalizing the results beyond Romania.

Therefore, the generalizability of these findings to non-Romanian populations is limited, as the results reflect the cultural, educational, and infrastructural particularities of the Romanian university context. Future cross-cultural studies are needed to explore whether similar fitness patterns occur in different countries with varying levels of physical activity support.

Moreover, while the EFB Activity Questionnaire evaluated current physical activity behavior, it did not collect information about participants’ exercise history during childhood or adolescence. This limitation may have influenced the interpretation of fitness outcomes and should be addressed in future retrospective or longitudinal research. Finally, the absence of follow-up data restricts the evaluation of the EFB system’s long-term impact on fitness development or behavioral change. Furthermore, the unequal distribution of participants between the two test profiles (TP1 and TP2), particularly the overrepresentation of females in TP1 and males in TP2, may have introduced imbalance in subgroup analyses, potentially influencing group comparisons. Finally, the absence of follow-up data restricts the evaluation of the EFB system’s long-term impact on fitness development or behavioral change and limits any conclusions regarding the effectiveness or predictive validity of the tool.

Future studies could consider using objective measures of physical activity, such as accelerometers or wearable trackers, to complement or validate self-reported classifications and minimize the risk of misclassification bias.

### 4.2. Strengths

The study employs the EFB, which comprehensively assesses key fitness components, alongside anthropometric measurements, providing a detailed evaluation of physical condition [28]. Unlike other widely used tools such as Eurofit or FitnessGram, the EFB protocol includes individualized feedback and categorizes participants into fitness profiles (TP1/TP2), which allows for more personalized and motivational fitness interventions.

The inclusion of 180 students from several academic programs enhances the breadth of the findings. This multidisciplinary sample enabled comparative subgroup analyses (e.g., sports vs. non-sports majors), increasing the applicability of the results to diverse university populations. By examining fitness levels in relation to demographic factors, the study offers a deeper understanding of physical fitness levels [3]. The use of ABSI in addition to BMI provides more accurate assessment of obesity-related health risks [51,72]. This dual-index approach allows for the identification of at-risk individuals who may be misclassified by BMI alone.

Altogether, these methodological features strengthen the study’s capacity to support targeted, data-driven strategies for fitness promotion in higher education settings.

## 5. Conclusions

This research highlights significant differences in physical fitness levels based on test profiles, with participants in the Advanced profile consistently outperforming those in the Basic profile. Additionally, Advanced profile participants exhibited lower BMI and ABSI, reinforcing the link between fitness and body composition. Sex-based differences were evident, with male participants achieving higher strength and coordination scores. Age-related analysis confirmed lower strength levels among older participants, consistent with existing cross-sectional research. Academic major also influenced fitness outcomes, with sports university students scoring higher in endurance, strength, and coordination compared to their peers from other areas.

These findings highlight significant differences in physical fitness among university students, shaped by factors such as test profile, sex, age, academic major, and body composition. The EFB served as a practical tool for classifying participants by fitness level, enabling structured assessment across groups. While this study did not evaluate the validity or reliability of the EFB instrument, its application in this context demonstrates its practical utility in academic settings. Given the high proportion of students classified in the Basic profile (TP1), these findings also highlight the effectiveness of the EFB in distinguishing between specific components of physical fitness and providing immediate, individualized feedback. This may help spark motivation for performance improvement among students. Furthermore, the results emphasize the importance of reinforcing physical education and sport participation, particularly within non-sport academic programs where fitness levels are markedly lower.

This study addresses a previously underexplored gap by applying the EFB to a university student population and examining its outcomes in relation to demographic and anthropometric factors. Its innovative contribution consists in offering a detailed, multidimensional characterization of health-related fitness among students, emphasizing how test profiles, body composition, sex, age, and academic background interact to shape physical fitness.

The results support our initial hypotheses, confirming that profile classification, sex, age, and academic specialization are relevant factors influencing physical performance in young adults. Our findings also have relevance for policy development. The EFB framework could be adapted to support national-level health strategies and Physical Activity Guidelines for Higher Education Institutions in Romania. By incorporating fitness assessment tools like the EFB into university curricula or institutional health programs, stakeholders could better monitor student health and implement targeted interventions for specific subgroups (e.g., students in sedentary majors or older age groups).

Future research should explore the long-term impact of EFB-based interventions and examine how integrating such tools into national education or public health policy might enhance population-level fitness awareness and behavior. In addition, follow-up studies should assess the predictive value of EFB performance for health outcomes, as well as validate its psychometric properties in diverse student populations.

## Figures and Tables

**Table 1:** Components, methods, and scoring criteria of the EFB assessment for TP1 (Basic) and TP2 (Advanced/Approved) levels.

Anthropometric Measurements/Questionnaires	Dimension and Items for TP1—Basic	Evaluation Method TP1	Dimension and Items for TP2—Advance/Approved	Evaluation Method TP2
a. Age b. Sex c. Weight d. Height e. Waist f. BMI g. ABSI h. Posture i. Physical Activity Questionnaire j. Health Questionnaire	**ENDURANCE** 1. Step test (max. 2, 40 min) or 2. Non-ex	1–3 points/test, according to scoring based on reference values	**ENDURANCE** 1. Step test (max. 6 min) or 2. Walking (2 km.)	1–5 points/test, according to age, sex
	**STRENGTH** 3. Simplified or normal push-up (rep.) 4. Standing up 5. Planking (30 s)		**STRENGTH** 3. Modified push-up (max. 40 s) 4. Jump and reach (cm) 5. Planking (max. 4 min)	
	**COORDINATION** 6. Jumping jack 7. Balancing one leg		**COORDINATION** 6. Walking backwards (6 m) 7. Flamingo balance (1 min)	
	**FLEXIBILITY** 8. Sit and reach		**FLEXIBILITY** 8. Sit and reach	
	**FINAL SCORES**	>7–11 points (according to age) = **BASIC**		>11–14 points = **ADVANCED** >15–20 points = **APPROVED**

Note: TP = Test Profile; TP1 = Basic Level; TP2 = Advanced/Approved Level; ABSI = A Body Shape Index; BMI = Body Mass Index; “Non-ex” = non-exercise estimation of endurance; Sit and Reach test evaluates lower back and hamstring flexibility; scoring is based on age- and sex-specific normative reference values; final classification: BASIC = 7–11 points, ADVANCED = 11–14 points, APPROVED = 15–20 points.

**Table 2:** Physical fitness score characterization (M ± SD), and comparison by profile (TP1 vs. TP2).

Parameter	Endurance	Strength	Flexibility	Coordination	Overall Fitness
TP1 (Basic)	2.80 ± 0.42	7.87 ± 0.99	2.64 ± 0.60	5.15 ± 0.55	10.86 ± 0.88
TP2 (Advanced)	3.00 ± 0.01	9.00 ± 0.01	2.72 ± 0.56	5.65 ± 0.72	12.66 ± 3.67
Total	2.90 ± 0.31	8.41 ± 0.90	2.68 ± 0.58	5.39 ± 0.68	11.73 ± 2.77
MD (B − A); CI95%	−0.19; [−0.28, −0.10]	−1.13; [−1.34, −0.92]	−0.08; [−0.25, 0.09]	−050; [−0.69, −0.32]	−1.80; [−2.58, −1.03]
TP1 vs. TP2 (*t*; *p*)	−4.260; 0.001 *	−10.610; 0.001 *	−0.907; 0.366	−5.263; 0.001 *	−4.588; 0.001 *

* Significant for *p* < 0.05; MD (B − A) = Mean Difference (Basic − Advanced); CI95% = Confidence Interval of 95%.

**Table 3:** Physical fitness comparison by sex, age, and academic major.

	Endurance	Strength	Flexibility	Coordination	Overall Fitness
Sex (*t*; *p*)	1.804; 0.073	6.754; 0.001 *	−1.109; 0.269	3.909; 0.001 *	−0.296; 0.767
Age (*F*; *p*)	0.974; 0.406	3.260; 0.023 *	1.244; 0.295	0.681; 0.565	1.037; 0.378
Major (*F*; *p*)	2.724; 0.015 *	7.640; 0.001 *	1.839; 0.094	2.702; 0.016 *	2.335; 0.034 *
MD (S − O); CI95%	0.12; [0.07–0.18]	0.72; [0.57–0.88]		0.40; [0.14–0.65]	1.54; [0.53–2.55]
ES (Cohen’s *d*)	0.644; moderate	1.331; large		0.754; moderate	0.622; moderate

* Significant for *p* < 0.05; MD (S − O) = Mean Difference (Sports major − Other academic majors); CI95% = Confidence Interval of 95%; ES = Cohen’s d.

**Table 4:** Combined effects of characteristics (TP, sex, age, major) with physical fitness parameters.

	Endurance	Strength	Flexibility	Coordination	Overall Fitness
TP * sex (*F*; *p*)	2.226; 0.138	8.575; 0.004 *	3.146; 0.078	0.920; 0.339	4.174; 0.043 *
TP * age (*F*; *p*)	0.024; 0.976	0.266; 0.767	0.099; 0.906	1.567; 0.212	0.370; 0.691
TP * major (*F*; *p*)	1.260; 0.291	2.370; 0.073	0.775; 0.510	0.780; 0.507	0.519; 0.670
Sex * age (*F*; *p*)	0.001; 1.000	0.001; 1.000	0.724; 0.396	0.080; 0.777	0.191; 0.663
Sex * major (*F*; *p*)	1.384; 0.234	1.651; 0.151	0.833; 0.528	1.774; 0.122	0.513; 0.766
Age * major (*F*; *p*)	1.493; 0.134	0.759; 0.692	0.874; 0.575	0.519; 0.900	1.021; 0.433

* Significant for *p* < 0.05.

**Table 5:** Association between the body composition and physical fitness of university students.

	Endurance	Strength	Flexibility	Coordination	Overall Fitness
BMI	−0.101; 0.177	−0.214; 0.004 *	0.005; 0.948	−0.277; 0.001 *	−0.175; 0.019 *
ABSI	−0.058; 0.440	−0.141; 0.060	−0.131; 0.081	0.075; 0.317	−0.264; 0.001 *
TP	0.304; 0.001 *	0.623; 0.001 *	0.068; 0.366	0.367; 0.001 *	0.325; 0.001 *
Sex	−0.134: 0.073	−0.452; 0.001 *	0.083; 0.269	−0.281; 0.001 *	0.022; 0.767
Age	−0.022; 0.769	−0.207; 0.005 *	0.101; 0.177	−0.028; 0.712	0.160; 0.032 *
Major	−0.057; 0.450	−0.213; 0.004 *	−0.046; 0.543	−0.130; 0.082	−0.149; 0.046 *

* Significant for *p* < 0.05.

## Data Availability

Data are contained within the article.
